# Ocular ketoconazole-loaded proniosomal gels: formulation, *ex vivo* corneal permeation and *in vivo* studies

**DOI:** 10.1080/10717544.2016.1247928

**Published:** 2017-02-06

**Authors:** Ghada A. Abdelbary, Maha M. Amin, Mohamed Y. Zakaria

**Affiliations:** 1 Department Pharmaceutics and Industrial Pharmacy, Faculty of Pharmacy, Cairo University, Cairo, Egypt and; 2 Department Pharmaceutics and Industrial Pharmacy, Faculty of Pharmacy, Sinai University, Sinai, Egypt

**Keywords:** Proniosomal gel, ketoconazole, ocular delivery, *ex vivo* corneal permeation, ocular keratitis

## Abstract

*Context*: Vesicular drug carriers for ocular delivery have gained a real potential. Proniosomal gels as ocular drug carriers have been proven to be an effective way to improve bioavailability and patient compliance.

*Objective*: Formulation and *in vitro*/*ex vivo*/*in vivo* characterization of ketoconazole (KET)-loaded proniosomal gels for the treatment of ocular keratitis.

*Materials and methods*: The effect of formulation variables; HLB value, type and concentration of non-ionic surfactants (Tweens, Spans, Brijs and Pluronics) with or without lecithin on the entrapment efficiency (EE%), vesicle size and *in vitro* KET release was evaluated. An *ex vivo* corneal permeation study to determine the level of KET in the external eye tissue of albino rabbits and an *in vivo* assessment of the level of KET in the aqueous humors were performed.

*Results and discussion*: *In vivo* evaluation showed an increase in bioavailability up to 20-folds from the optimum KET proniosomal gel formula in the aqueous humor compared to drug suspension (KET-SP). The selected formulae were composed of spans being hydrophobic suggesting the potential use of a more hydrophobic surfactant as Span during the formulation of formulae. Factors that stabilize the vesicle membrane and increase the entrapment efficiency of KET (namely low HLB, long alkyl chain, high phase transition temperature) slowed down the release profile.

*Conclusions*: Proniosomal gels as drug delivery carriers were proven to be a promising approach to increase corneal contact and permeation as well as retention time in the eye resulting in a sustained action and enhanced bioavailability.

## Introduction

The use of vesicular systems is considered as an alternative way to overcome many problems associated with ocular drug delivery and to enhance the topical controlled delivery of ophthalmic drugs with respect to traditional eye drops. The limited extent of ocular absorption caused by physiological constraints, such as induced lacrimation, normal tear turnover and rapid precorneal clearance, leading to a significant drug loss is still remaining the main challenge facing ocular drug delivery system (Lee & Robinson, 1986). That is also the reason of the limited bioavailability of ophthalmic solutions, where the therapeutic effect is achieved by daily frequent instillation of the solution. Nevertheless, the systemic absorption of the drug drained through the nasolacrimal duct systems can cause side effects and the drastic damage in the ocular surface both are resulted from the frequent use of concentrated solutions (Topalkara et al., 2000; Kaur et al., 2004).

Absorption of drugs into the eye requires good corneal penetration and a prolonged contact time with the corneal tissue. Various attempts were investigated for prolonging the contact time between the drug and corneal–conjunctival epithelium in order to increase its bioavailability. In spite of increasing the corneal–conjunctival contact time by adopting various delivery systems, certain disadvantages were noted, including poor patient compliance, side effects such as blurring of vision, sticky sensation and induced reflex blinking due to irritating properties (Kaur et al., 2004).

In vesicular drug delivery systems, the drug is encapsulated in lipid vesicles, which can cross cell membrane. Vesicles, therefore, as drug carriers can enhance both the bioavailability and the disposition of the drug. Vesicular systems provide prolonged duration of action at the corneal surface by preventing ocular metabolism by enzymes in the lacrymal fluid (Allam et al., 2011). Vesicular drug delivery systems used in ophthalmic preparations broadly include liposomes and niosomes. Niosomes have gained popularity in ocular drug delivery research and area potential delivery system for the effective treatment of glaucoma (Paul et al., 2010) and various other conditions. Allam et al. (2011) reported that acyclovir-loaded niosomes were effective for the treatment of herpes simplex keratitis, a condition that can lead to blindness. Similarly, gentamicin-loaded niosomes provided controlled, opthalmic delivery (Abdelbary & El-Gendy, 2008) and brimonidine-loaded niosomes were therapeutically effective with a long duration of action due to slow and prolonged zero-order release of drug (Prabhu et al., 2010).

Proniosomal gels are liquid crystalline (gel) vesicular structures produced from nonionic surfactants having the ability to entrap both hydrophilic and lipophilic drugs, they can promote adherence to the corneal/conjunctival surface when used as ophthalmic preparations. They can be transformed easily into niosomes immediately upon hydration (Gupta et al., 2007). Recently, various studies proved the reliability of proniosomal gels in promoting the ocular bioavailability of different drugs. Ocular proniosomal gels of lomefloxacin HCl were prepared using different types of nonionic surfactants solely and as mixtures with Span 60 in order to improve its ocular bioavailability for the management of bacterial conjunctivitis (Khalil et al., 2016). Tacrolimus-loaded proniosomes containing poloxamer 188 and lecithin as surfactants, cholesterol as a stabilizer and minimal amount of ethanol were prepared and characterized regarding the occurrence of corneal allograft rejection and the median survival time of corneal allografts (Li et al., 2014).

Many advantages of proniosomes are presented over niosomes, namely physical stability (avoiding aggregation, fusion, leakage), shielding for the entrapped drug from hydrolysis (Hu & Rhodes, 2000). The ease of preparation of proniosomes by simply dissolving the surfactant in the least amount of organic solvent (Vora et al., 1998) can solve the problems arising from other tedious preparation methods of niosomes such as reversed-phase evaporation and ether or ethanol injection methods (Weiner, 1994).

Ketoconazole (KET) is abroad spectrum antifungal, highly lipophilic molecule (log *p* = 4), belonging to imidazoles, its absorption is highly dependent on gastric pH and its oral administration causes many side effects (nausea, vomiting, gastrointestinal disturbance, hepatitis, gynecomastia and adrenal cortex suppression) (O’Brien, 1999). The mode of action of KET is to inhibit synthesis of ergosterol and to increase fungal cellular permeability. Ketoconazole (KET) has been used for various types of ocular fungal species such as Aspergillus species, Candida species and some Fusarium species (Zhang et al., 2008).

The topical use of KET is characterized by a short residence and ocular half-life (Zhang et al., 2008). In spite of KET lipophilicity, its high molecular weight (531.44 Da) offers an obstacle against its transport across the biological membrane, so passage through corneal stroma is hampered. Therefore, a suitable carrier system for KET is required (Barar et al., 2008). Furthermore, it is desired to attain a high concentration of KET crossing cornea reaching the posterior segment of the eye for an effective treatment of ocular fungal infections; fungal keratitis, candidal chorioretinitis that are caused by *Candida albicans* (Ahuja et al., 2008).

Some trials were performed aiming to incorporate the fungistatic molecule KET into polymeric (Eudragit®RS 100) and solid lipid (Gelucire®44/14) nanoparticles by quasi-emulsion solvent evaporation technique aiming ocular application (Demirel & Genc, 2015). Solid lipid nanoparticles (SLN) ocular dispersion of KET comprising Compritol 888 ATO and PEG 600 matrix were prepared using hot high-pressure homogenization (Kakkar et al., 2015).

*In vivo* behavior of proniosomes-derived niosomes showing advantages as drug carriers, comprising lower cost and toxicity, easy storage and handling as well as increased stability. Encapsulation of drug in niosomal formulations reduces the toxicity in various therapies and applications and also prolongs the encapsulated drug circulation time and changes drug distribution in the body. Niosomes as drug delivery vesicles increases absorption of some drugs through cell membranes and cellular uptake via endocytosis and so confines the drug in tissues and targeted organs (Yasam et al., 2014).

Based on the aforementioned, the aim of this work is to improve the ocular bioavailability of KET through the preparation of ocular KET mucoadhesive proniosomal gels using different types of nonionic surfactants (Spans, Tweens, Brijs and Pluronics) with or without lecithin. Furthermore, the prepared proniosomal gel formulae were characterized regarding encapsulation efficiency percent (E.E%), vesicle size analysis and *in vitro* drug release. An *ex vivo* corneal permeation study of the selected gel formulae was performed to determine the level of KET in the external eye tissue of albino rabbits after topical application. Finally, an *in vivo* characterization of the optimum proniosomal gel formula was performed through evaluating the level of KET in the aqueous humors of thirty-six albino rabbits using a validated HPLC method.

## Materials and methods

### Materials

Ketoconazole (KET) powder was kindly supplied by El Nile pharmaceutical company (Cairo, Egypt). Span 20, Span 60, Span 65, Span80, Tween 80, Brij 35, Brij 72, Brij 92, Pluronic F68, Pluronic L121, L-α-phosphatidylcholine (PC) from egg yolk, Cholesterol (CH) powder were purchased from Sigma Chemicals Co. (St. Louis, MO). Methanol, sodium chloride, potassium chloride, sodium bicarbonate, calcium chloride, sodium hydroxide, magnesium chloride, potassium dihydrogen orthophosphate and absolute ethanol were purchased from El-Nasr Chemical Co. (Cairo, Egypt). Spectra/Pore® dialysis membrane (12 000–14 000 molecular weight cutoff) was purchased from Spectrum Laboratories Inc (Los Angeles, CA).

### Preparation of KET proniosomal gels

The composition of the different prepared KET proniosomal gels is shown in Table 1. Proniosomal gels were prepared by the coacervation-phase method previously reported by (Mishra et al., 2012) with some modifications. In this method, the accurately weighted amount of drug, surfactant cholesterol/or lecithin (as permeation enhancer) were transferred to tightly closed glass vials to which absolute ethanol (0.4 mL) was added. The vials were transferred to water bath at (55–60 °C) for 5 min with continuous shaking till complete dissolution of cholesterol. To each of the formed transparent solutions, about 0.15 mL of hot distilled water (55–60 °C) was added while keeping in water bath for 3–5 min till a clear or translucent solution was produced. The mixtures were allowed to cool down to room temperature.

**Table 1.** Composition of the different prepared ketoconazole-loaded proniosomal gels.

Formula	Span20	Span60	Span65	Span80	Tween80	Brij35	Brij72	Brij92	Pluronic (F68)	Pluronic (L121)	Lecithin
F1	500 mg										
F2	250 mg										250 mg
F3		500 mg									
F4		250 mg									250 mg
F5			500 mg								
F6			250 mg								250 mg
F7				500 mg							
F8				250 mg							250 mg
F9					500 mg						
F10					250 mg						250 mg
F11						500 mg					
F12						250 mg					250 mg
F13							500 mg				
F14							250 mg				250 mg
F15								500 mg			
F16								250 mg			250 mg
F17									500 mg		
F18									250 mg		250 mg
F19										500 mg	
F20										250mg	250 mg

### Hydration step and formation of niosomes

Niosomes were prepared by hydration of the previously prepared proniosomal gels as described by Mokhtar et al., (2008). About 7 mL of Sorensen’s phosphate buffer (pH 7.4) was added to a certain weight (100 mg) of the gel from each vial followed by heating at a temperature of 40–50 °C for approximately one minute with the aid of Vortex (Maxi mix, M 36710 mixer, Barnstead International, Dubuque, IA). The final volume was adjusted to 10 mL using Sorensen’s phosphate buffer (pH 7.4).

### *In vitro* characterization of the prepared KET proniosomal gels

#### Entrapment efficiency (EE%)

The entrapment efficiency of KET from the prepared niosomes was calculated following ultra-centrifugating of 1 mL of the niosomal suspension in Sorensen’s phosphate buffer (pH 7.4) at 15 000 rpm for one hour using cooling centrifuge at 4 °C (Beckman, Fullerton, Canada). The niosomes were separated from the supernatant, washed twice, with 1 mL Sorensen’s phosphate buffer each time and recentrifuged again for 30 min. The amount of entrapped KET was determined after lysis of the separated vesicles by sonication (Model 275 T (Crest Ultrasonics Corp., Trenton, NJ) with methanol; (Maestrelli et al., 2005). The concentration of the entrapped drug was determined spectrophotometrically (Shimadzu, model UV-1601 PC, Kyoto, Japan) at λ_max_ 295 nm against methanol as blank.

The percent entrapment efficiency was calculated as follows:

(1) $$\mathrm{KET}\;\%\mathrm{EE}\;=\;\frac{\mathrm{amount}\;\mathrm{of}\;\mathrm{KET}\;\mathrm{entrapped}}{\mathrm{total}\;\mathrm{amount}\;\mathrm{of}\;\mathrm{KET}}\;\times \;100$$

#### Vesicle size measurement

The vesicle size of the prepared KET niosomes after hydration was determined by light scattering based on LASER diffraction using the Malvern Mastersizer X LASER scattering instrument (detection limit 0.1–2000 μm) (Malvern Instruments Ltd., Worcestershire, UK) (Agarwal et al., 2001).

#### In vitro release of KET from the prepared proniosomal gels

The *in vitro* release of KET from the prepared proniosomal gels was determined using the membrane diffusion technique; (Junyaprasert & Manwiwattanakul, 2008). A certain weight (200 mg) of the prepared proniosomal gel equivalent to 2 mg KET was transferred to a glass cylinder having the length of 10 cm and diameter of 2.5 cm fitted at its lower end with presoaked cellulose membrane on which the gel was spread over (Spectra/Pore dialysis membrane 12 000–14 000 Mwt cutoff). The glass cylinder was attached to the shaft of the dissolution apparatus and then suspended in the dissolution flask of a USP dissolution apparatus (VK 7000 Dissolution Testing Station, Vankel Industries, Inc., NJ) containing 100 mL simulated lacrimal fluid (SLF, pH 7.4) kept at a temperature of 37 ± 0.5 °C (Abdelbary et al., 2008). The glass cylinder was allowed to rotate at a constant speed (25 rpm). For comparison, the *in vitro* release of an equivalent amount of KET suspension (KET-SP) was carried out adopting the same procedure as previously described.

At predetermined time intervals (1, 2, 3, 4, 6, 8, 12 and 24 h), aliquots were withdrawn and the drug content was determined spectrophotometrically at 295 nm, the mean values of three runs (± SD) were calculated.

Based on the above tests, selection of KET proniosomal gel formulae was based on the highest E.E%, with the lowest *in vitro* release at Q8hrs. The selected formulae were further investigated through *ex-vivo* corneal permeation.

### *Ex vivo* corneal permeation of the selected KET proniosomal gels

*Ex vivo* corneal permeability study of the selected KET proniosomal gels had been approved by Cairo University Research Ethics Committee. Permeability studies were performed using Franz diffusion cell consisting of two-limbed reservoir (Aggarwal et al., 2004) having a donor compartment of about 15 mm orifice diameter with effective diffusion area of 0.78 cm^2^ and a receptor volume (7 mL). The isolated cornea together with each of the selected KET proniosoma gel formulae was mounted on one limb, and the other limb was used as the sampling port. The permeation study was maintained at a constant temperature (37 ± 0.2 °C) using a magnetic stirrer (Wisestir magnetic stirrer, China).

Rabbit corneas used in this study were separated from male New Zealand albino rabbits. Rabbits were killed by injection of an overdose of sodium phenobarbital and the corneas were excised from the globes. The cornea used in the experiment was immersed in simulated lacrimal fluid (pH 7.4) for 30 min before the conduction of the experiment in order to simulate the same physiological conditions of the eye. Extreme care was taken not to produce any wrinkles or folding of the membrane before mounting the cornea onto the ring of the diffusion apparatus. A dose of 50 μL of KET-proniosomal gel from the selected proniosomal gel formulae (F2, F3, F4, F5 and F6) was placed on the isolated cornea in 5 mL freshly prepared simulated lacrimal fluid (pH 7.4). Aliquots of the medium were withdrawn from the sampling port after specified time intervals; 1, 2, 4, 6, 8 and 10 h and were replaced with equal volume of fresh medium to maintain a constant volume.

Samples were analyzed using a validated HPLC method (Zhang et al., 2008). The mobile phase consisted of a mixture of potassium dihydrogen phosphate (0.02 mol/L) aqueous solution and methanol in the ratio of (25:75 v/v) (pH was adjusted to 3.0 using phosphoric acid) with a flow rate of 1.5 mL/min. The determination was performed at 235 nm using HPLC instrument (Hitachi LaChrome Elite, Tokyo, Japan).

HPLC instrument was equipped with a model series L-2000 organizer box, L-2300 column oven, L-2130 pump with built in degasser, Rheodyne 7725i injector with a 20 μL loop and a L-2455 photodiode array detector (DAD), separation and quantitation were made on a 250 × 4.6 mm (i.d.), 5 μm ODS column (Inertsil, Tokyo, Japan). The HPLC was operated by EZ chrom Elite version 3.3.2 SP1 by Agilent.

### *In vivo* study of the optimum proniosomal gel formula

#### Determination of KET level in aqueous humors of rabbits

The *in vivo* characterization of the optimum proniosomal gel formula (F2) was performed through evaluating the level of KET in the aqueous humors of 36 albino rabbits. This was accomplished by comparing the level of KET from the optimum proniosomal gel formula with 1% KET ophthalmic suspension (KET-SP), respectively, after topical application.

Thirty-six healthy New Zealand albino male rabbits, weighing about 2.0–3.0 kg, were divided randomly into two groups (18 rabbits in each group). The animals were housed in standard cages, in a light-controlled room at 20 ± 1 °C and 50 ± 5% relative humidity, with no restriction of food or water. During the experiments, the rabbits were placed in restraining boxes, where they could move their eyes and heads freely. All experiments were carried out under veterinary supervision, used in full compliance with local, national, ethical and regulatory principles for animal care. Each group was divided into six subgroups corresponding to withdrawal time intervals (three rabbits in each subgroup). The rabbits were kept under anesthesia throughout the experiment using sodium pentobarbital (30 mg/kg) injected into the marginal ear vein.

KET-proniosomal gel was applied to one group of animals whereas KET-SP was instilled into the eyes of the second group. A dose of (50 μL) of KET-proniosomal gel or KET-SP was instilled into the lower cul-de-sac of the eye of each rabbit. Aqueous humor was withdrawn with a 26-G needle attached to a tuberculin syringe at 0.5, 1, 2, 4, 6 and 8 h. Samples were stored until they were extracted. The extraction was performed in accordance to (Zhang et al., 2008), First, 100 μL of aqueous humor was transferred into a glass test tube and 100 μL of phosphate buffer saline (PBS) was added. The mixture was vortexed and 2 mL of dichloromethane was added. The mixture was vortexed for 2 min and then centrifuged at 3000 rpm for 5 min.

The upper layers were aspirated and discarded, and the organic layer was transferred to the other cone glass test tube. The organic layer was evaporated to dryness. The residue was reconstituted in a 100 μL of mixture of methanol and deionized water (50:50 v/v). The mixture was vortexed for 1 min and centrifuged at 4000 rpm for 15 min. Then, 20 μL aliquot of each supernatant was directly injected into HPLC to be analyzed. No internal standard was required as the peaks were separated from that of aqueous humor and no noise or overlapping occurred.

#### Occular irritancy test

An ocular irritancy testing was also performed in order to verify the safety of the optimum proniosomal gel. The potential ocular irritancy and/or damaging effects of the tested proniosomal gel formula (F2) was evaluated by observing any redness, inflammation or increased tear production, upon application to the eyes of albino rabbits. The formulation was tested on three albino rabbits. The experiment was performed by a single instillation (50 μL) of the proniosomal preparation under test into the conjunctival sac of one eye, while the contralateral eye served as control. Both eyes of the rabbits under test were examined for any sign of irritation, such as conjunctival corneal edema and/or hyperhemia upon direct visual observation using a slit lamp, before treatment and 1, 8 and 24 h following drug instillation (Colo et al., 2001).

## Results and discussion

### Preparation of proniosomal gels

Proniosomal gel formulae of KET were prepared efficiently adopting coacervation-phase method using different types of nonionic surfactants as; Spans (sorbitan fatty acid esters), Tweens (polyoxyethylene sorbitan esters), Brijs (polyoxyethylene alkyl ethers) and Pluronics (polyoxyethylene-polyoxypropylene block copolymers) with or without lecithin in a ratio of S.A.A:lecithin (1:1 w/w) together with a constant amount (50 mg) of cholesterol per each formulation (Table 1).

Nonionic surfactants are the most common type of surface-active agents used in preparing vesicles due to their superior benefits with respect to stability, compatibility and toxicity. They are generally less toxic, less hemolytic and less irritating to cellular surfaces and tend to maintain near physiological pH in solution (Kumar & Rajeshwarrao, 2011). Cholesterol must be added to the surfactant in order to form a bilayered vesicle, also cholesterol enhances the stability of the prepared vesicles. The addition of cholesterol enables more hydrophobic surfactants to form vesicles, suppresses the tendency of the surfactant to form aggregates and provides greater stability to the lipid bilayer by promoting the gel liquid transition temperature of the vesicle (Lawrence et al., 1996).

Lecithin is generally named depending on its source of origin such as soya lecithin from soya beans and egg lecithin from egg yolk. Phosphatidyl choline is such a major component of lecithin. In the vesicular system, it plays a number of important roles: (a) it acts as permeation enhancer; (b) enhances the percent drug entrapment due to high *T*_c_ (phase transition temperature); (c) leads to vesicles of smaller size due to increase in hydrophobicity which results in the reduction of vesicle size; (d) prevents the leakage of drug (Rawat et al., 2011).

Finally, the addition of water leads to swelling of bilayer which is due to the interaction between water and the polar groups of the surfactants leading to the formation of multivesicular, multilamellar and spherical shaped structures (Rawat et al., 2011).

### Entrapment efficiency (EE%)

#### Effect of surfactant HLB

Table 2 shows the entrapment efficiency (EE%± SD) of the different prepared KET proniosomal gel formulae. It is clear that results ranged from 37.50 ± 1.15 to 93.00 ± 1.10%. Regarding formulae prepared using different grades of Spans (F1-F8), The significantly highest (*p *<* *0.05) EE% (93.00 ± 1.10%) was obtained from formula F6 prepared using Sp 65 (HLB = 2.1) and lecithin in a ratio of (Sp 65: lecithin 1:1 w/w). It is clear that the EE% from formulae prepared using different grades of Spans followed the order of: Sp 65 > Sp60 > Sp20 > Sp 80.

**Table 2.** Physical evaluation of the prepared ketoconazole-loaded proniosomal gels.

Formula	%Drug entrapped ± S.D	Z-average (d.nm)±S.D	PDI ± S.D
F1	57.90 ± 2.90	1322 ± 82	0.09 ± 0.00
F2	87.10 ± 1.19	590.80 ± 34	0.14 ± 0.06
F3	85.20 ± 1.67	855 ± 57	0.49 ± 0.13
F4	86.60 ± 1.80	535.20 ± 28	0.15 ± 0.08
F5	83.80 ± 2.30	339 ± 19	0.61 ± 0.18
F6	93.00 ± 1.10	559.10 ± 22	0.60 ± 0.20
F7	47.00 ± 1.25	2695 ± 98	0.61 ± 0.10
F8	79.50 ± 3.10	413.60 ± 24	0.11 ± 0.03
F9	37.50 ± 1.15	97.56 ± 12	0.37 ± 0.11
F10	49.50 ± 2.34	140.60 ± 10	0.21 ± 0.09
F11	40.60 ± 1.55	4432 ± 105	1 ± 0.42
F12	49.90 ± 1.25	452.70 ± 63	0.49 ± 0.11
F13	60.60 ± 1.21	2271 ± 79	1 ± 0.37
F14	74.90 ± 0.65	550 ± 72	1 ± 0.50
F15	62.90 ± 2.25	1096 ± 89	1 ± 0.39
F16	72.70 ± 2.96	1140 ± 99	0.34 ± 0.13
F17	41.60 ± 1.31	1961 ± 88	0.87 ± 0.29
F18	51.70 ± 2.96	805 ± 63	0.36 ± 0.09
F19	51.40 ± 3.85	663.40 ± 92	0.77 ± 0.34
F20	70.70 ± 2.70	510.30 ± 49	0.62 ± 0.23

It is known that the head groups are similar in all Spans, while the alkyl hydrocarbon chains are different. In spite of the same head groups and same carbon atoms (C18) in the alkyl chain of Sp 60 (HLB = 4.7) and Sp 80 (HLB = 4.3) having almost the same HLB value, they differ in the structure of the alkyl chain. The presence of double bonds in the alkyl chains of Sp 80 leads to a markable increment in the permeability of bilayer of niosomes, thus possibly justifying the lower entrapment efficiency of Sp 80 formulations; 47.00 ± 1.25 and 79.50 ± 3.10 for F7 and F8, respectively (Table 2). Surfactants of longer saturated alkyl chains showed higher entrapment efficiency (Guinedi et al., 2005).

Increasing the alkyl chain number leads to an increase in entrapment efficiency. It is known that Sp 65 has three alkyl chains (stearate alkyl chain) with an HLB value of 2.1. Consequently, it may play a role in decreasing the permeability of the membrane and increases the encapsulation efficiency. Similar results were obtained by (Hao et al., 2002) who reported that the lower the HLB of the used surfactant, the higher the entrapment efficiency of colchicine within the prepared niosomes.

The HLB values of Sp 60, Sp 65 and Sp 80 are equal to 4.7, 2.1 and 4.3, respectively, compared to 8.6 in case of Sp 20. The fact that lower HLB spans exhibit the highest E.E% was attributed to many reasons such as; being solid at room temperature with higher phase transition temperature (*T*_c_), the higher (*T*_c_) of surfactants, they are more involved in a more rigid bilayers highly ordered gel formation, leading to a higher entrapment efficiency.

The gel transition temperature of spans increases as the length of the alkyl chain increases. Thus, sorbitan monolaurate (Sp20) (C12) is liquid at room temperature (*T*_c_ = 16 °C); sorbitan monostearate (Sp60) (C18) has a gel transition temperature of 54 °C and about 53 °C for sorbitan tristearate (Sp65) (C54) chain (Bouwstra et al., 1997). In addition, the lowest transition temperature of Sp 80 (C18) (*T*_c_ = 12 °C) among all tested Spans was the main reason of its lowest E.E% among other spans (Kibbe, 2000). As possessing the highest phase transition temperature (*T*_c_) in Spans provides the highest entrapment for the drug and *vice versa* as lower (*T*_c_) surfactants are more prone to form less packed ordered liquid form (Hao et al., 2002).

In the current study, the E.E% of formulae prepared using T80 surfactant (F9–F10) showing 37.50 ± 1.15 and 49.50 ± 2.34 of KET entrapped for F9 and F10 which were significantly less (*p *<* *0.05) than their corresponding ones (F7–F8) having 47.00 ± 1.25 and 79.50 ± 3.10, respectively, using Span surfactant having the same alkyl chain length (Sp 80). T80 being hydrophilic surfactant with high HLB value of 15 compared to 4.3 in case of the hydrophobic Sp 80, this probably elucidates the lower entrapment efficiency of T80 compared to Sp 80 formulations as previously discussed (Hao et al., 2002).

Brij surfactants are polyoxyethylene alkyl ethers that differ in the number of hydrophilic oxyethylene groups and length of hydrophobic alkyl chain. Regarding the E.E% of proniosomal gel formulae prepared using different grades of Brijs (F11-F16); Brij 35 (polyoxyethylene (23) lauryl ether), Brij 72 (polyoxyethylene (2) stearyl ether) and Brij 92 (polyoxyethlene (2) oleyl ether) having the corresponding HLB values of 16.9, 4.9 and 5, respectively. According to the results shown in Table 2, both long-chain surfactants (C18), namely Brij 72 and Brij 92, with HLB value of approximately five had significantly (*p *<* *0.05) higher E.E% compared to that of Brij 35 (C12). This could be attributed to the increased bilayer hydrophobicity due to their longer alkyl chains and lower HLB values leading to effective encapsulation of the drug within the hydrophobic core of the bilayer (Abdelbary & Aburahma, 2012).

Concerning proniosomal gel formulae (F17–F20) prepared using different grades of Pluronics (F68 and L121) which belong to a group of surfactants formed of triblock copolymers composed of a central hydrophobic polyoxypropylene (POP) fragment and similar hydrophilic chains of polyoxyethylene (POE) on either sides. Variation of the length of each of the blocks enables the modulation of the copolymer properties (Abdelbary & Aburahma, 2012).

Pluronic F68 is composed of 75 POE units and 30 POP units, while Pluronic L121 consists of two POE units and 4500 POP units (Moghimi & Hunter, 2000) with an HLB of 25 and 0.5, respectively. The entrapment efficiencies of KET within the niosomal vesicles prepared using Pluronics were significantly different (*p *<* *0.05) as the lower HLB L121 gave rise to a significantly higher drug entrapment of: 51.40 ± 3.85 and 70.70 ± 2.70 for F19 and F20 prepared with Pluronic L121 compared to 41.60 ± 1.31 and 51.70 ± 2.96 for F17 and F18 using Pluronic F68 respectively. Pluronics are suggested to stabilize the lipid membranes of the vesicles in the presence of cholesterol by adsorption on the membrane and through selective incorporation into low lipid density regions of the membrane, holding lipid molecules to pack firmly on the vesicles phospholipid membrane retarding the drug leakage (Wu et al., 2004).

Based on the above results, vesicles formation ability of nonionic surfactant depends on its structure and hydrophilic–lipophilic balance which are considered as good indicators of the entrapping efficiency of any surfactant and its vesicle-forming ability.

The critical packing parameter CPP = (*v/lc ao*) of a given surfactant depends on the balance between the critical hydrophobic group length (*l*c), hydrophobic group volume (*v*) and the area of the hydrophilic head group (*a*o) (Uchegbu & Vyas., 1998). A value of CPP lying between 0.5 and 1 indicates that the surfactant is more prone to form vesicles. A value of CPP below 0.5 indicates the spherical micelle formation and a CPP of surfactant above one would lead to inverted micelles formation (Uchegbu & Florence., 1995).

It was reported that Both Sp 65, Brij 72 and Pluronic L121 were able to form vesicular structure with high entrapment efficiency even in the presence of low cholesterol concentration because they have relatively large hydrophobic moieties with low water solubility (Manosroi et al., 2003). On the other hand, other Span grades, Brij 35, Tween 80 and Pluronic F68 were not able to form niosomes in the presence of small amounts of cholesterol, this might be attributed to their high HLB values, solubilizing property and therefore micelle formation ability that dissolves the small amounts of cholesterol (Pardakhaty et al., 2007), this might also explain the low entrapment ability of the proniosomal formulae prepared by these surfactants.

#### Effect of cholesterol

In order to enhance drug-loading capacity, cholesterol content should be increased during the preparation of niosomal systems. Also there is a great influence on vesicle stability and permeability upon addition of cholesterol (Gregoriadis, 1993). The influence of changing cholesterol ratio within the lipid composition on KET entrapment efficiency was determined. It was found that changing surfactant:cholesterol ratio from 10:1 to 5:1 led to a significant increase in E.E% as reported by (El-Laithy et al., 2011). Furthermore, (Mohammed & Perrie., 2005) studied the effect of cholesterol incorporation into liposomes on the entrapment efficiency of the poorly soluble drug ibuprofen. It was suggested that increasing cholesterol leads to the enhancement in drug loading capacity but upon exceeding a certain limit, a great reduction in drug incorporation occurred, this might be due to two conflicting factors:

1. With increasing cholesterol, the lipophilicity and permeability of the bilayer decreased and rigidity increased leading to the lipophilic drug to be trapped efficiently into bilayers as vesicles formed.
2. In contrast, higher amounts of cholesterol may compete with the drug for filling in the space within the bilayer. It was suggested that decreasing the entrapment efficiency with increasing cholesterol ratio above certain limit may be due to the distruption in the regular linear structure of vesicular membranes occurred on increasing cholesterol beyond a certain concentration. In addition, the ratio of cholesterol may influence the ability of proniosomal gel formation. These results were in accordance with (Ibrahim et al., 2008) who found that there is no ability to form proniosomal gels in the presence of Sp 20 and Sp 80 at cholesterol concentration less than 20% being liquids at room temperature. Other surfactants such as; Brij 35, Brij 92, Tween 80 and Pluronic F68 require also a higher concentration of cholesterol which might be attributed to their high HLB values, solubilizing property, leading to micelle formation that dissolves the small amount of cholesterol (Yoshioka et al., 1994).

On the other hand, proniosomal gels can be produced in case of Brij 72 (HLB 4.9, *T*_c_ = 44 °C), Sp 60 (HLB 4.7, *T*_c_ = 54 °C), Sp 65 (HLB 2, *T*_c_ =53 °C) even at low cholesterol content as they are solids at room temperature (Uchegbu & Vyas, 1998). Moreover, below transition temperature, cholesterol made the membrane less ordered and increasing cholesterol has been found to increase membrane fluidity to the extent where the phase transition is abolished (Arunothayanun et al., 2000).

#### Effect of lecithin

Results shown in Table 2 revealed that the addition of lecithin generally led to a significant increase in E.E% in all the prepared KET proniosomal gel formulae. This might be due to its high *T*_c_ (phase transition temperature), decrease in membrane permeability therefore preventing drug leakage, hence the increase in KET content within the prepared vesicles.

### Vesicle size analysis

The mean particle size and size distribution of the freshly prepared hydrated niosomes are demonstrated in Table 2. It can be noted that the vesicle size ranged from 97.56 ± 12 nm to 4432 ± 105 nm indicating that the average particle size of the measured hydrated niosomal suspension varies from the nanometer to submicron range. The particle size distribution of all the tested formulae demonstrated unimodal normal symmetrical frequency distribution patterns (PDI ≤ 1).

The prepared proniosomal gels will help in ocular delivery by improving corneal permeation, prolonging ocular mean residence time, thus increasing corneal contact time with the formula, it has been reported that ophthalmic preparations should have particle size less than 10 000 nm in order not to cause ocular irritation (Hecht, 2001). Regarding ocular drug delivery systems, smaller particles are characterized by greater surface area available for conjugation between the cornea and proniosomal gel formula (Yoncheva et al., 2005).

Furthermore, the prepared nano to submicron vesicles will help in crossing the ocular physiological and anatomical constraints. Kassem et al. (2007) reported that as the particle size in the drug suspension decreased, this leads to an increase in the mean residence time of drugs on the ocular surface. Therefore, a smaller size could be an added advantage for treatment of superficial fungal infections. Upon variation of amounts of proniosomal ingredients, the following effects on vesicle size were observed. Increasing surfactant/lipid ratio led to an increase in vesicle size and was attributed to the increase in the overall degree of hydrophilicity. Also, the increase in mean vesicle size by increasing lecithin content can be also accepted if we considered the long hydrocarbon chain of lecithin molecules (18 °C). The opposite held true with increasing the cholesterol amount that was associated with a decrease in the hydrophilicity of bilayers, thus limiting the water intake to the vesicles core and resulted in a subsequent decrease in mean vesicle size. Finally, the increase in mean vesicle size with increasing drug load was attributed to the drug entrapped in the hydrophobic domain of the vesicle, causing the bilayer molecules to become apart from each other leading to an increase in vesicle size (Hathout et al., 2007).

Concerning formulae (F1–F8) prepared using different grades of Spans (Sp20, Sp60, Sp65 and Sp80), it is clear that the largest vesicle size was obtained from F1 (Sp20) and F7 (Sp80) having an average diameter of 1322 ± 82 and 2695 ± 98 nm together with lowest E.E% of 57.90 ± 2.90 and 47.00 ± 1.25% respectively. Both formulae (F1 and F7) were composed of S.A.A: lecithin ratio of 10:1 w/w, it is clear that decreasing lecithin content will decrease the hydrophobicity and hence contributed to larger vesicle size. There is an inverse relationship between particle size and E.E% in proniosomes prepared with different Span derivatives.

Regarding proniosomal gel formulae prepared using the hydrophilic T80 (F9–F10) (HLB = 15), on contrary to Sp80 (HLB = 4.3), the smallest the particle size of 97.56 ± 12 and 140.60 ± 10 nm for F9 and F10 compared to 2695 ± 98 and 413.60 ± 24 for F7 and F8 containing the hydrophobic Sp80. A direct relationship correlates the particle size and EE% in case of T80, as the vesicles size might depend on the properties of the molecules entrapped in the hydrophobic area of the vesicle bilayer. The vesicle size depends on the distance between the bilayers, which increased due to the inclusion of drug molecules within (Hathout et al., 2007).

The average vesicle size of proniosomal gel formulae prepared using different grades of Brijs was >1000 nm except for the two formulae F12 and F14, having 452.70 ± 63 and 550 ± 72 nm respectively. According to the results shown in Table 2, both long-chain surfactants (C18), namely Brij 72 and Brij 92, with HLB value of aproximate 5 had significantly (*p *<* *0.05) higher E.E% compared to that of Brij 35 (C12) as previously discussed. The presence of high lecithin ratio in both F12 and F14 contributes in further reduction of hydrophobicity resulting in smaller vesicle size.

From Table 2, it is clear that the mean average vesicle size of proniosomal gel formulae prepared using different Pluronic grades; Pluronic F68 and L121 with an HLB of 25 and 0.5 respectively varied according to the HLB of the given surfactant, as increasing the hydrophobicity attributes to a decrease in free energy resulting in smaller vesicles.

### *In vitro* KET release

**Figure 1.**
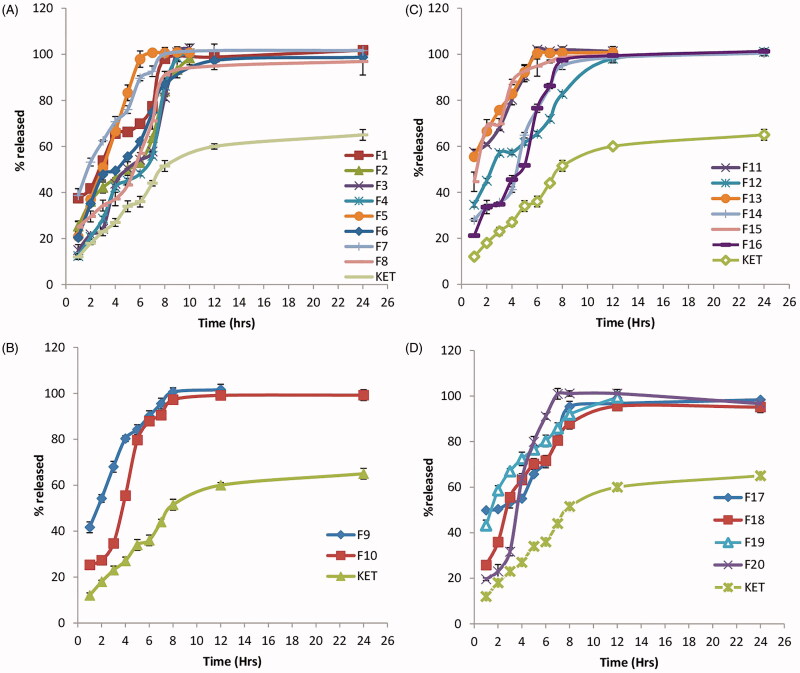
*In vitro* release profile of the different prepared KET-loaded proniosomal gel formulae prepared with: (A) Spans (B) Tween 80 (C) Brijs (D) Pluronics.

**Figure 2.**
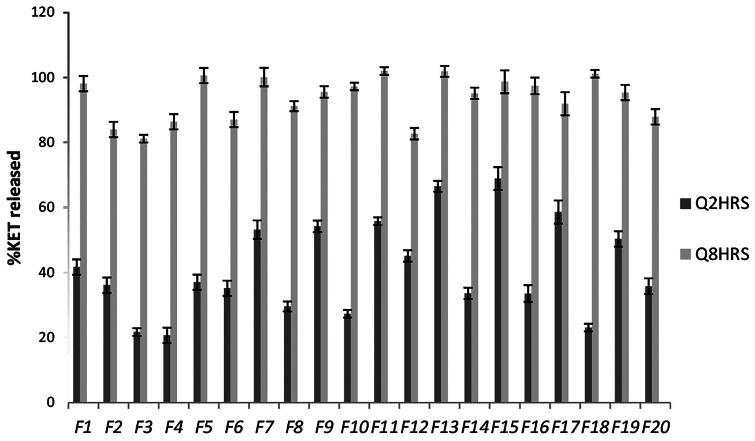
*In vitro* release of the different prepared KET-loaded proniosomal gel formulae at Q 2 h and Q 8 h.

Results of *in vitro* release of KET from the different prepared proniosomal gels are shown in Figure 1(A–D). The percentage of KET released from the different prepared proniosomal gels after 2 h (Q 2 h) and 8 h (Q 8 h) are shown in Figure (2). It is clear that KET release after 2 h (Q 2 h) ranged from 20.7%± 0.15 to 68.5% ± 4.7, while the release after 8 h (Q 8 h) ranged from 81.16% ± 1.178 to 101.97% ± 1.2, respectively.

The release profiles of KET from the different prepared proniosomal gel formulae were found to be biphasic release as reported by Mokhtar et al. (2008). A rapid drug leakage was observed in the initial phase, where about 25–55% of the entrapped drug was released within the first few hours of proniosomal incubation in 100 mL of simulated lacrimal fluid (pH 7.4). While in the second phase, a slow release of KET was observed from the different proniosomal formulations. The initial rapid phase might be due to the unentrapped drug, which is mainly present between the large hydrocarbon chains in the lipid bilayers of proniosomal vesicles which leads to a rapid leakage from the vesicles in large simulated lacrimal fluid (pH 7.4) until reaching equilibrium. Moreover, it has been reported that, this drug explosion occurs as a result that the highly ordered lipid particles cannot accommodate large amounts of drug (Wissing et al., 2004).

Accordingly, factors that stabilize the vesicle membrane and increase the entrapment efficiency of a hydrophobic drug such as KET (namely low HLB, long alkyl chain, high phase transition temperature) will slow down the release profile. The release profiles of the proniosomes revealed a significant increase (*p *<* *0.05) in the percentage drug released with the increase in HLB since hydrophilic surfactants have higher solubilizing power on hydrophobic solutes in aqueous medium compared to hydrophobic (Pardakhaty et al., 2007). Increasing lecithin content resulted in an increase in phase transition temperature (*T*_c_) together with a more intact lipid bilayer with low permeability which hindered drug leakage leading to a significant slow release profile of entrapped drug from the vesicles (*p *<* *0.05) compared to lecithin-free proniosomes (Guinedi et al., 2005; AbdElbary et al., 2008).

The addition of cholesterol (50 mg) in the preparation of KET proniosomal gel formulations resulted in further decrease in KET release due to the decrease in leakage and permeability of niosomal vesicular membrane in the presence of cholesterol. (Cocera et al., 2003) suggested that the presence of cholesterol resulted in an optimum lipophilicity which decreased the formation of the transient hydrophilic holes by the incorporation of cholesterol, this was done by decreasing membrane fluidity, responsible for drug release through liposomal layers.

Based on the previous results, Formulae (F2, F3, F4, F5 and F6) were selected for further investigations, having E.E% of not less than 80%, mean vesicle size less than 1000 nm with P.D.I value less than 1 and less than 40% KET released within the first 2 h. It is clear that all the selected formulae were composed of spans being hydrophobic compared to other used surfactants (Tween, Brijs and Pluronics) suggesting the potential use of a more hydrophobic surfactant as Span during the formulation of proniosomal gel formulae.

### *Ex vivo* corneal permeation

**Figure 3.**
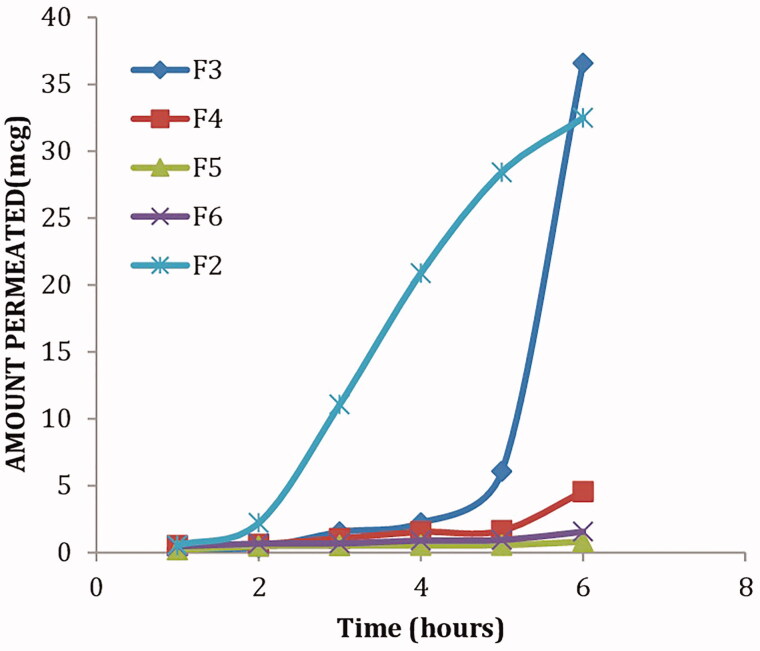
*In vitro* corneal permeation of the selected KET-loaded proniosomal gel formulae.

Figure (3) shows the cumulative amounts of KET permeated from the selected proniosomal gel formulations through isolated corneal rabbit as described before. It is clear that a significant higher amount (*p < *0.05) of KET permeated from formulae F2 and F3 compared to other formulae (F4, F5 and F6). Furthermore, F2 and F3 showed the highest steady-state flux and permeability coefficient as shown in Table 3. It is clear that proniosomes prepared using Sp65 (F5, F6) showed the least amount of KET permeated, this might be attributed to the transition temperature of the used surfactant, where the high transition temperature of Sp65 (53 °C) made the proniosomes in a more packed ordered gel state at the specified permeation temperature (37 °C) (Vora et al., 1998). On the other hand, the lower transition temperature of Sp20 (16 °C) in formula (F2) allows the proniosomes to be in a completely fluid state at the specified permeation temperature. F2 was selected as the optimum formula showing significantly higher (*p <* 0.05) permeability coefficient and steady state flux of 0.000244 cm^2^/h and 2.44 mcg/cm^2^h, respectively.

**Table 3.** Permeability parameters of the selected ketoconazole-loaded proniosomal gels.

Formula	Permeability coefficient (cm^2^/h)	Steady-state flux (mcg/cm^2^hr)	Correlation coefficient (*R*)
F2	0.000244	2.44	0.993
F3	0.0002009	2.009	0.99
F4	2.36 × 10^−5^	0.236	0.91
F5	3.18 × 10^−6^	0.0318	0.88
F6	6.39 × 10^−6^	0.0639	0.924

### *In vivo* study of the optimum proniosomal gel formula

#### Determination of KET level in aqueous humor

**Figure 4.**
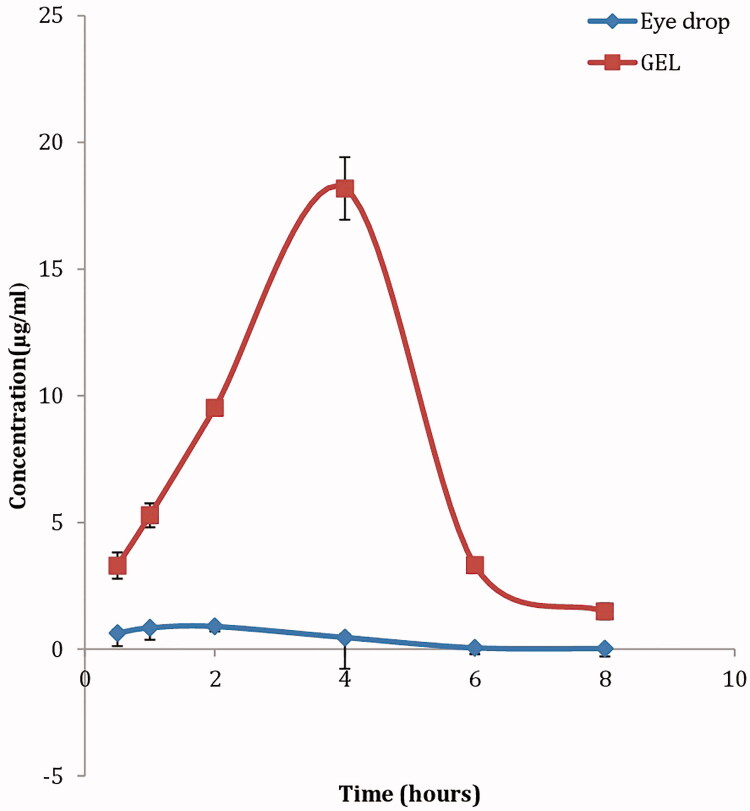
Concentration-time curve of KET-proniosomal gel formula (F2) and KET-SP in aqueous humor of albino rabbits.

Based on the aforementioned results, formula F2 composed of 250 mg span 20, 250 mg lecithin together with 50 mg cholesterol) was selected for *in vivo* evaluation; where KET levels in the aqueous humor were determined following topical application of KET-proniosomal gel (F2) and KET-SP at different intervals (Figure 4). Table 4 shows the different pharmacokinetic parameters (*C*_max_, *T*_max_, AUC, *k*, MRT and *T*_1/2_) calculated for both formula and drug suspension. Following topical application of KET-Gel (*C*_max_, 18.8 μg/mL) was attained after 4 h which was 22 times greater than that of KET-SP (*C*_max_, 0.896 μg/mL) reached after 2 h, respectively. The KET concentrations in aqueous humor post-6 and 8 h instillation of KET-Gel were 66 and 73 times higher than that of KET-SP, respectively, with about 20 fold increase in KET-proniosomal gel (F2) bioavailability in the aqueous humor over KET-SP (Table 4).

**Table 4.** Pharmacokinetic parameters of ketoconazole after topical administration of KET-Gel optimum formula and KET-SP rabbit's eye.

Pharmacokinetic parameters	KET-Gel (F2)	KET-SP
*C*_max_ (mcg/ml)	18.8 ± 1.23	0.896 ± 0.052
*T*_max_ median *t*_max_ (h)^a^	4	2
AUC 0–8 (mcg.h/mL)	60.1 ± 3.04	3.15 ± 0.27
AUC 0–∞ (mcg.h/mL)	62.48 ± 3.56	3.18 ± 0.25
*K* (h^−1^)	0.625 ± 0.045	0.682 ± 0.251
*T*_1/2_ (h)	1.1 ± 0.07	1.01 ± 0.264
MRT (h)	3.88 ± 0.1	2.5 ± 0.0305

Topical administration of KET-Gel to rabbits with an intact epithelium resulted in a significant increase (*p *<* *0.05) in KET level in cornea exceeding the minimum inhibitory concentration (MIC) of ocular isolates of fungi; Filamentous fungi and Yeast, whose MIC (<0.8 μg/mL) (Therese et al., 2006). The elevated KET levels in the cornea and aqueous humor following the administration of KET-Gel might be due to the increase in the amount of KET dissolved in the precorneal area leading to high concentration gradient, favoring good permeation, together with higher contact time with the corneal area.

#### Ocular irritancy test

The ocular irritancy testing revealed that the tested formula KET-Gel (F2) did not show any sign of redness, inflammation or increased tear production over the study period (24 h), Therefore, it could be concluded that the proniosomal gel formua (F2) was nonirritant following topical application into the eye.

## Conclusions

The results of this study showed that the type of surfactant, HLB and lecithin content altered the entrapment efficiency and KET release rate of the prepared proniosomal gel formulae. It could be concluded that proniosomal gel formulations loaded with KET showed prolonged ocular action and higher bioavailability than formulations containing KET in non-niosomal form. Therefore, proniosomal gel may be considered as a promising ophthalmic drug delivery system of KET for the topical treatment of ocular Keratitis.
